# Error analysis in the determination of the electron microscopical contrast transfer function parameters from experimental power Spectra

**DOI:** 10.1186/1472-6807-9-18

**Published:** 2009-03-26

**Authors:** Carlos Oscar S Sorzano, Abraham Otero, Estefanía M Olmos, José María Carazo

**Affiliations:** 1 Escuela Politécnica Superior, Universidad San Pablo-CEU, Campus Urb. Montepríncipe s/n, E-28668 Boadilla del Monte, Madrid, Spain; 2 Biocomputig Unit of the National Center of Biotechnology (CSIC), Madrid, Spain

## Abstract

**Background:**

The transmission electron microscope is used to acquire structural information of macromolecular complexes. However, as any other imaging device, it introduces optical aberrations that must be corrected if high-resolution structural information is to be obtained. The set of all aberrations are usually modeled in Fourier space by the so-called Contrast Transfer Function (CTF). Before correcting for the CTF, we must first estimate it from the electron micrographs. This is usually done by estimating a number of parameters specifying a theoretical model of the CTF. This estimation is performed by minimizing some error measure between the theoretical Power Spectrum Density (PSD) and the experimentally observed PSD. The high noise present in the micrographs, the possible local minima of the error function for estimating the CTF parameters, and the cross-talking between CTF parameters may cause errors in the estimated CTF parameters.

**Results:**

In this paper, we explore the effect of these estimation errors on the theoretical CTF. For the CTF model proposed in [1] we show which are the most sensitive CTF parameters as well as the most sensitive background parameters. Moreover, we provide a methodology to reveal the internal structure of the CTF model (which parameters influence in which parameters) and to estimate the accuracy of each model parameter. Finally, we explore the effect of the variability in the detection of the CTF for CTF phase and amplitude correction.

**Conclusion:**

We show that the estimation errors for the CTF detection methodology proposed in [1] does not show a significant deterioration of the CTF correction capabilities of subsequent algorithms. All together, the methodology described in this paper constitutes a powerful tool for the quantitative analysis of CTF models that can be applied to other models different from the one analyzed here.

## Background

The transmission electron microscope distorts the structural information contained in the electron micrographs by changing the amplitude of the Fourier coefficients at all spatial frequencies and flipping their phase at certain annular regions [2]. This effect is usually modeled in Fourier space by the Contrast Transfer Function (CTF), which in turn has to be estimated from the electron micrographs. Normally, a theoretical model of the CTF is assumed and the parameters defining this model are optimized so that the experimentally observed PSD and the theoretically predicted PSD coincide as much as possible [1,3-10]. Therefore, the PSD has to be estimated first. This step is traditionally performed by the fast, although less accurate, periodogram averaging [10-13] or parametric methods, more accurate but much slower to compute [8,12]. The estimated periodogram can be further enhanced [14] to highlight the Thon rings and, therefore, facilitate the task of fitting the parameters of the theoretical model.

Fully two-dimensional models multiply by three the number of parameters needed since each parameter is allowed to vary in two dimensions. For instance, the defocus is assumed to vary elliptically, thus three parameters are needed for its full description (major axis, minor axis, and the angle between the major axis and the coordinate horizontal axis); the same applies to all parameters varying in 2D. Moreover, rich physical models like the ones in [1,9,10] need many parameters to account for a pletorah of physical effects. Finally, as shown in [1], rich two-dimensional background models are needed to fully account for the astigmatism introduced not only by the electron microscope but also by the film scanner.

Overall, theoretical CTF models may end up with many parameters demanding robust optimization algorithms that avoid local minima. Cross-talking between parameters cannot be avoided, ie, sometimes the same CTF can be obtained with two different sets of CTF parameters. Moreover, the amount of noise present in the electron micrographs passes to the PSD estimates, no matter how much averaging is performed, and errors in the estimates of the CTF parameters are to be expected. Sensitivity analysis [15] is a branch of mathematics studying how errors at the input of a mathematical model translate into errors at its output.

In this paper, we propose to use sensitivity analysis to identify those CTF parameters that have the strongest influence in the estimation on the CTF. Knowing this list of "sensitive" parameters, simpler models for the CTF can be proposed (as will be shown in the Results Section, two parameters of the CTF model analyzed can be safely removed). We also propose the use of bootstrap resampling to estimate the accuracy in the estimation of each individual parameter and to reveal the internal structure of the model: which model parameters influence in a given parameter, for instance, which are the model parameters influencing the defoci? The bootstrap resampling also allows us to estimate the experimental distribution of each CTF parameter. Confidence intervals for each CTF parameter can be computed using these experimental distributions. We use these confidence intervals to identify which parameters are not significantly different from zero, and therefore can be omitted from the CTF model. Finally, we use Factor Analysis in order to further clarify the internal structure of the CTF model (thanks to this analysis, the CTF parameters can be divided into different groups, each one accounting mainly for a different part of the CTF). In this work we apply the general principles of sensitivity analysis to the identification of the most relevant parameters in the CTF model introduced by [1] as well as their internal relationships and accuracy of their estimates.

Moreover, we explore the effect of the variability in the estimation of the CTF parameters in subsequent algorithms for CTF correction. In particular, we analyzed its effects on CTF phase correction and CTF amplitude correction using the Iterative Data Refinement (IDR) [16]. We show that in our experiments, the estimation errors of the CTF detection performed in [1] does not significantly deteriorates the CTF correction.

## Results and discussion

As described in the Methods section, the average sensitivity of the CTF with respect to a given parameter (, Eq. 21) is a measure of how variations in that parameter translate into variations of the CTF. In a similar manner we also define the average sensitivity of the PSD and the average sensitivity of the first zero of the CTF with respect to a given parameter. In the following sections we present and discuss our results.

### Results

To estimate the sensitivity of the CTF on the model parameters we used two sets of experimental images (LTag and GltS) corresponding to samples embedded in ice with no carbon. The LTag images correspond to the Large T antigen [17,18], while the GltS images correspond to the Glutamate synthase [19]. The LTag images had a sampling rate of 5.6 Å per pixel while the GltS images were digitized with a pixel size of 1.59 Å. The two datasets used in this paper are the same ones used in [1]. In all, we studied the sensitivity of the parameters using a total of 217 micrographs. The rationale for employing two distinct datasets is not to bias the statistical analysis by using a single type of micrographs. The fact that both datasets have very different sampling rates helps in the analysis of the effect of the sampling rate. The average sensitivity of the CTF with respect to a given parameter (Eq. 21) was evaluated as follows. For each micrograph and parameter, the CTF was studied with the value estimated by the CTF fitting program () [1]. Then, we perturbed each parameter individually by a small amount () as described in the Methods section in order to test its influence in the CTF. In particular, we studied variations of -20%, -10%, -5%, -2%, -1%, 1%, 2%, 5%, 10%, and 20% (the expected value in Eq. 21 was computed for each variation and the resulting sensitivities were averaged). For a particular variation of the parameter (), we computed the integral in Eq. 21. The set of all perturbations and all micrographs empirically defined the statistical distribution of the sensitivity over which an ensemble average was taken.

The analysis carried out in the "Sensitivity analysis" Section for the CTF can be analogously be performed on the theoretical PSD (Eq. 1) simply by replacing *H *in that section by *P S D*_*theoretical*_. The corresponding sensitivity will be referred to as . In the same way, the sensitive analysis can also be performed on the first zero of the CTF (that is the frequency of the first sign change of the CTF; since the CTF is a 2D function, we will measure the sensitivity of the average of the first zero along all possible directions in 2D). The corresponding sensitivity of this average first zero will be referred to as . Summarizing, the CTF sensitivity is analyzed according to three different measures: the average sensitivity of the CTF itself as is computed in the "Sensitivity analysis" Section (); the average sensitivity of the theoretical PSD (); and the average sensitivity of the frequency of the first zero along all possible directions (). The CTF sensitivity results are summarized in Table 1. In each case, the sensitivity values are arranged in decreasing order.

**Table 1:** CTF Sensitivity

					
*V*	61.23 (100.00)	*s*_*m*_	0.4890 (100.00)	*V*	0.2901 (100.00)
Δ*f*_*M*_	37.93 (61.95)	*s*_*M*_	0.4613 (94.34)	Δ*f*_*M*_	0.2507 (86.42)
Δ*f*_*m*_	37.45 (61.16)	*b*	0.2447 (50.04)	Δ*f*_*m*_	0.2497 (86.07)
*C*_*a*_	6.132 (10.01)	*K*_*G*_	0.2441 (49.92)	*Q*_0_	0.0331 (11.41)
Δ*V/V*	5.32 (8.69)	*V*	0.0371 (7.59)	*θ*	0.0053 (1.83)
*θ*	1.51 (2.47)	Δ*f*_*m*_	0.0288 (5.89)	*T*_*m*_	0.0010 (0.34)
*Q*_0_	0.96 (1.57)	Δ*f*_*M*_	0.0285 (5.83)	*C*_*s*_	0.0001 (0.03)
*α*	0.80 (1.31)	*K*	0.0096 (1.96)	Δ*V/V*	NA
*C*_*s*_	0.78 (1.27)	*C*_*a*_	0.0061 (1.25)	*C*_*a*_	NA
K	0.72 (1.18)	Δ*V/V*	0.0058 (1.19)	*K*	NA
Δ*R*	0.24 (0.39)	*K*_*s*_	0.0037 (0.76)	*α*	NA
Δ*F*	0.03 (0.05)	*C*_*m*_	0.0025 (0.51)	Δ*R*	NA
*T*_*m*_	NA	*K*_*g*_	0.0022 (0.45)	Δ*F*	NA
		*G*_*M*_	0.0021 (0.43)		
		*C*_*M*_	0.0019 (0.39)		
		*G*_*m*_	0.0018 (0.37)		
		*Q*_0_	0.0015 (0.31)		
		*θ*_*G*_	0.0013 (0.27)		
		*c*_*m*_	0.0013 (0.27)		
		*α*	0.0011 (0.22)		
		*g*_*M*_	0.0010 (0.20)		
		*g*_*m*_	0.0008 (0.16)		
		*θ*	0.0007 (0.14)		
		*c*_*M*_	0.0004 (0.08)		
		Δ*R*	0.0002 (0.04)		
		*θ*_*s*_	0.0002 (0.04)		
		*C*_*s*_	0.0001 (0.02)		
		*θ*_*g*_	0.0001 (0.02)		
		Δ*F*	NA		
		*T*_*m*_	NA		

Each column represents the average error sensitivity of the CTF (*S*_*H*_), the theoretical PSD (*S*_*PSD*_), and the first zero of the CTF (*S*_*zero*_). Columns are sorted by descending sensitivity. In parenthesis is shown the percentage of the corresponding sensitivity with respect to the maximum sensitivity in that column. Those entries marked as NA mean that the parameter is not affecting the measured quantity.

The highest values of sensitivity are found in . Interestingly, within this measure, the most significant parameter is the microscope voltage (*V*) that is a user supplied parameter. Not surprisingly, the energy spread of the electrons () is a related magnitude and is also a parameter with a high sensitivity. The next two most sensitive parameters are the defoci and the chromatic aberration.

Considering , parameters from the background *PSD *(*s*_*m*_, *s*_*M*_, *b *and *K*_*G*_) are by far the most sensitive. Of the CTF parameters, only the microscope voltage and the defoci have a significant weight. As expected, in the case of , the most sensitive values defining the first zero of the CTF are the microscope voltage *V*, the defoci and the fraction of electrons being scattered *Q*_0_.

We obtain a relative sensitivity of each parameter dividing each sensitivity column by its maximum value and multiplying by 100 (data shown in Table 1). Adding all relative sensitivities (data shown in Table 2) we can have an idea of which are the most important model parameters regarding the three quantities studied in this article (CTF, *PSD *and first zero of the CTF). By far the most important parameters are the microscope voltage and the defoci. Next, we find four parameters defining the background PSD (*s*_*m*_, *s*_*M*_, *b *and *K*_*G*_). Finally, we have the fraction of electrons being scattered *Q*_0 _(a parameter affecting the first zero of the CTF) and the chromatic aberration (a parameter mostly affecting the CTF envelope decay). The previous results seems to point out that there are "redundant" or less important parameters in our PSD model. To test up to which degree this statement is true, we randomly chose one of the micrographs and estimated several simplified PSD models. The full model has 29 parameters out of which 27 have to be estimated (the microscope voltage *V *and its spherical aberration *C*_*s *_are assumed to be given). The different simplified models proceed by removing parameters from the full model following an order indicated by ascending sensitiveness: less sensitive parameters are removed first (however, they are removed in "blocks" of related parameters). We explored 3 different simplified models:

**Table 2:** CTF Overall sensitivity and accuracy

**Parameter**	**Symbol**	**Overall Sensitivity**	**Accuracy (%)**
Microscope voltage	*V*	207.59	NA
Major defocus	Δ*f*_*M*_	154.19	1.18
Minor defocus	Δ*f*_*m*_	153.13	1.07
Background PSD	*s*_*m*_	100.00	5.50
Background PSD	*s*_*M*_	94.34	4.38
Background PSD	*b*	50.04	5.26
Background PSD	*K*_*G*_	49.92	11.91
Fraction of scattered electrons	*Q*_0_	13.28	21.86
Chromatic aberration	*C*_*a*_	11.26	14.29
Energy spread	Δ*V/V*	9.87	52.95
Defocus azimuthal angle	*θ*	4.44	NA
CTF Gain	*K*	3.14	7.41
Aperture semiangle	*α*	1.53	54.23
Spherical aberration	*C*_*s*_	1.33	NA
Background PSD	*K*_*s*_	0.76	14.70
Background PSD	*C*_*m*_	0.51	40.61
Background PSD	*K*_*g*_	0.45	11.22
Focal plane displacement	Δ*R*	0.43	NA
Background PSD	*G*_*M*_	0.43	23.58
Background PSD	*C*_*M*_	0.39	38.18
Background PSD	*G*_*m*_	0.37	29.72
Sampling rate	*T*_*m*_	0.34	NA
Background PSD	*c*_*m*_	0.27	40.60
Background PSD	*θ*_*G*_	0.27	NA
Background PSD	*g*_*M*_	0.20	17.51
Background PSD	*g*_*m*_	0.16	13.29
Background PSD	*c*_*M*_	0.08	19.07
Perpendicular displacement	Δ*F*	0.05	NA
Background PSD	*θ*_*s*_	0.04	NA
Background PSD	*θ*_*g*_	0.02	NA

The overall sensitivity column is the sum for each parameter of the percentages shown in Table 1. The accuracy column shows the accuracy in the estimation of each parameter as estimated by bootstrapping (see text).

1. Simplified model 1: Since the CTF is usually computed on non-drifted images and as shown by the sensitivity analysis the PSD is not very sensitive to the drift parameters (Δ*F *and Δ*R*), our simplified model 1 does not estimate these two parameters and sets them to 0. This model has a total of 25 parameters to be estimated.

2. Simplified model 2: The last step of the PSD estimation is the computation of the subtractive Gaussian parameters (*K*_*g*_, *c*_*M*_, *c*_*m*_, *θ*_*g*_, *g*_*M *_and *g*_*m*_). In our experience this last Gaussian helps to accurately fit the low pass frequencies. However, as shown by the sensitive analysis, the theoretical PSD is not too sensitive to these parameters, so we also estimated a simplified model without this last Gaussian (and without the parameters already removed in the Simplified model 1). There is a total of 19 parameters to be estimated.

3. Simplified model 3: In this simplified model we forced the remaining Gaussian (the one with positive sign in the background PSD) to be symmetric (*G*_*M *_= *g*_*m*_, *C*_*M *_= *C*_*m *_and *θ*_*G *_= 0) besides all the simplifications already done in the Simplified model 2. This leaves only 16 parameters to be estimated.

**Figure 1:**
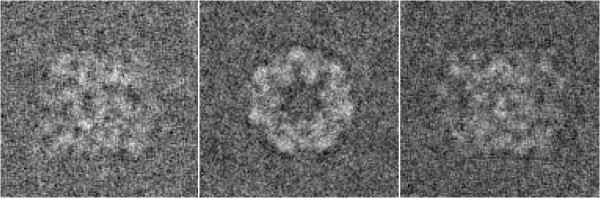
**Projection examples**. Sample projection images of GroEL used for the CTF correction experiment.

Figure 1 shows the results of the PSD fitted by the full model and the simplified models. The goal function of the Simplified model 1 was 0.1% larger than that of the full model, and the goal function of the Simplified models 2 and 3 were 0.7% larger. Due to the decrease in the number of parameters to be estimated, there was a time saving of 3%, 14% and 19% respectively. As can be seen there is no significant visual difference between the Full model and the Simplified model 1, but there is a significant difference between the Full model and the Simplified models 2 and 3. As discussed in the next section, the difference is caused by the suppression of parameters which are not too sensitive but are significantly different from zero (in the order of thousands).

To evaluate the estimation accuracy of each of the model parameters, the bootstrap resampling strategy described in the "Accuracy of the CTF estimates" Section was followed. One thousand random samples where extracted from the dataset of a single micrograph of the LTag group. All fitted models had the same user-supplied parameters (microscope voltage, sampling rate and spherical aberration). Due to the results in our previous experiment, we removed from the model the perpendicular and focal plane displacements. After solving for the corresponding one thousand regression problems, the ensemble of all model parameters were collected. 3.6% of these regression parameters were considered as failures of the algorithm to correctly estimate the model parameters and the corresponding models were deleted from the dataset. The accuracy of an estimate was measured as the ratio between the median of absolute deviations (MAD, a robust equivalent of the standard deviation) and the median of the absolute value of the parameter being considered (a robust equivalent of the mean). Working with medians is a robust way of estimating the central position of a distribution. The accuracy of the value of the goal function being minimized in [1] of the remaining 97.4% bootstrapped samples was 0.5%. This low value indicates that the remaining bootstrapped models were quite homogeneous with respect to the regression error. The accuracy of each parameter was estimated and the resulting values are listed in Table 2. Those entries with NA indicate that the accuracy was not available in this case because the parameter is supplied by the user, or the parameter has not been estimated (displacements), or the parameter is meaningless in this case (the image used for the example was not astigmatic and therefore the angles of the ellipses involved in the model can take any value).

The analysis of the empirical distribution of each parameter computed by the bootstrap showed that the confidence interval of 95% of none of the parameters (except the azimuthal angle) included the zero value. This means that we can reject the hypothesis with a 95% confidence level that any of the parameters was really null, and therefore the corresponding parameter could have been removed from the regression. The bootstrapped ensemble also allows the estimation of pairwise correlation coefficients. Tables 3 and 4 show for each model parameter the set of parameters which are significantly correlated to it with a confidence of 99%. The tables also show the corresponding correlation coefficients. It can be seen that the different coefficients can be grouped in subgroups of large correlations. To identify these subgroups we used factor analysis. We used ten factors for the decomposition and kept only the first seven since their associated eigenvalue was larger than 1. Table 5 shows those factor loadings greater than 0.5 for each factor (ie, those CTF parameters that correlate more than 0.5 with the factor). It can be seen how the factor loadings create a partition of the model parameters into subgroups that are strongly correlated among each other. In the Discussion Section we propose an interpretation of each factor in terms of the different aspects of the CTF (different kinds of envelopes, the ideal CTF, and the different frequency ranges of the background PSD).

**Table 3:** Correlation of each model parameter with the rest of model parameters

**Parameter**	**Symbol**	**Correlated parameters**
Major defocus	Δ*f*_*M*_	*Q*_0 _(-0.87), Δ*f*_*m *_(0.86), *b *(0.15), *K*_*g *_(-0.19), *c*_*m *_(0.13), *c*_*M *_(0.10), *g*_*m *_(-0.17), *g*_*M *_(-0.10), *K*_*s *_(-0.15), *s*_*m *_(-0.15), *s*_*M *_(-0.11)
Minor defocus	Δ*f*_*m*_	*Q*_0 _(-0.88), Δ*f*_*M *_(0.86), *b *(0.21), *K*_*g *_(-0.22), *c*_*m *_(0.18), *c*_*M *_(0.10), *g*_*m *_(-0.17), *g*_*M *_(-0.10), *K*_*s *_(-0.21), *s*_*m *_(-0.20), *s*_*M *_(-0.16)
Background PSD	*s*_*m*_	*K*_*s *_(0.96), *s*_*M *_(0.98), *b *(-0.74), *K*_*G *_(-0.67), *C*_*M *_(0.31), *G*_*M *_(-0.31), *G*_*m *_(-0.26), *K*_*g *_(0.19), *c*_*m *_(-0.16), *g*_*M *_(0.27), *g*_*m *_(0.11), *Q*_0 _(0.11), Δ*f*_*m *_(-0.20), Δ*f*_*M *_(-0.15), *α *(0.20), *K *(0.12)
Background PSD	*s*_*M*_	*K*_*s *_(0.95), *s*_*m *_(0.98), *b *(-0.67), *K*_*G *_(-0.62), *C*_*M *_(0.27), *G*_*M *_(-0.30), *G*_*m *_(-0.26), *K*_*g *_(0.22), *c*_*m *_(-0.19), *g*_*M *_(0.28), Δ*f*_*m *_(-0.16), Δ*f*_*M *_(-0.11), *α *(0.22), *K *(0.19)
Background PSD	*b*	*K*_*s *_(-0.83), *s*_*m *_(-0.74), *s*_*M *_(-0.67), *K*_*G *_(0.47), *C*_*m *_(0.13), *G*_*M *_(0.55), *G*_*m *_(0.49), *K*_*g *_(-0.23), *c*_*m *_(0.14), *g*_*M *_(-0.15), *g*_*m *_(-0.10), Δ*f*_*m *_(0.21), Δ*f*_*M *_(0.15), *α *(-0.12)
Background PSD	*K*_*G*_	*K*_*s *_(-0.67), *s*_*m *_(-0.67), *s*_*M *_(-0.62), *b *(0.47), *C*_*M *_(-0.58), *C*_*m *_(-0.35), *G*_*M *_(-0.13), *c*_*M *_(-0.33), *α *(-0.12)
Fraction of scattered electrons	*Q*_0_	Δ*f*_*m *_(-0.88), Δ*f*_*M *_(-0.87), *K*_*g *_(-0.29), *c*_*m *_(-0.29), *c*_*M *_(-0.22), *g*_*m *_(0.16), *G*_*m *_(0.17), *G*_*m *_(0.14), *c*_*M *_(0.12), *K*_*s *_(0.12), *s*_*m *_(0.11)
Chromatic aberration	*C*_*a*_	Δ*V/V *(-0.71)
Energy spread	Δ*V/V*	*C*_*a *_(-0.71), *K *(0.19), *α *(0.14)
CTF Gain	*K*	*α *(0.74), Δ*V/V *(0.19), *s*_*M *_(0.19), *s*_*m *_(0.12), *K*_*g *_(0.10), *g*_*m *_(-0.11)
Aperture semiangle	*α*	*K *(0.74), Δ*V/V *(0.14), *K*_*s *_(0.20), *s*_*M *_(0.22), *s*_*m *_(0.20), *K*_*g *_(0.30), *K*_*G *_(-0.12), *b *(-0.12)
Background PSD	*K*_*s*_	*s*_*m *_(0.96), *s*_*M *_(0.95), *b *(-0.83), *K*_*G *_(-0.67), *C*_*M *_(0.27), *G*_*M *_(-0.34), *G*_*m *_(-0.29), *K*_*g *_(0.31), *c*_*m *_(-0.24), *g*_*M *_(0.23), Δ*f*_*m *_(-0.21), Δ*f*_*M *_(-0.15), *α *(0.20), *Q*_0 _(0.12)
Background PSD	*C*_*m*_	*K*_*G *_(-0.35), *C*_*M *_(0.68), *G*_*M *_(0.60), *G*_*m *_(0.56), *b *(0.13), *K*_*g *_(-0.20), *c*_*M *_(0.21), *g*_*M *_(0.15)
Background PSD	*K*_*g*_	*c*_*M *_(-0.60), *c*_*m *_(0.55), *K*_*s *_(0.31), *s*_*M *_(0.22), *s*_*m *_(0.19), *b *(-0.23), *K *(0.10), *α *(0.30), *Q*_0 _(0.29), Δ*f*_*M *_(-0.19), Δ*f*_*m *_(-0.22), *C*_*m *_(-0.20), *G*_*M *_(-0.10),
Background PSD	*G*_*M*_	*K*_*G *_(-0.13), *C*_*M *_(0.66), *C*_*m *_(0.60), *G*_*m *_(0.86), *b *(0.55), *K*_*s *_(-0.34), *s*_*M *_(-0.30), *s*_*m *_(-0.31), *K*_*g *_(-0.10), *c*_*m *_(-0.11) *Q*_0 _(0.14)
Background PSD	*C*_*M*_	*K*_*G *_(-0.58), *C*_*m *_(0.68), *G*_*M *_(0.66), *G*_*m *_(0.66), *K*_*s *_(0.27), *s*_*M *_(0.27), *s*_*m *_(0.31), *c*_*M *_(0.21), *c*_*m *_(-0.17), *g*_*M *_(0.27), *g*_*m *_(0.14) *Q*_0 _(0.13)
Background PSD	*G*_*m*_	*C*_*M *_(0.66), *C*_*m *_(0.56), *G*_*M *_(0.86), *b *(0.49), *K*_*s *_(-0.29), *s*_*M *_(-0.26), *s*_*m *_(-0.26), *c*_*m *_(-0.17), *Q*_0 _(0.17)
Background PSD	*c*_*m*_	*K*_*g *_(-0.55), *c*_*M *_(0.71), *g*_*m *_(0.31), *g*_*M *_(0.27), *b *(0.14) *K*_*s *_(-0.24), *s*_*M *_(-0.19), *s*_*m *_(-0.16), *C*_*M *_(-0.17), *G*_*M *_(-0.11), *G*_*m *_(-0.17), *Q*_0 _(-0.29), Δ*f*_*m *_(0.17), Δ*f*_*M *_(0.13)
Background PSD	*g*_*M*_	*c*_*M *_(0.34), *c*_*m *_(0.27), *g*_*m *_(0.71), *b *(-0.15) *K*_*s *_(0.23), *s*_*M *_(0.28), *s*_*m *_(0.27), *C*_*M *_(0.27), *c*_*m *_(0.15), Δ*f*_*m *_(-0.10), Δ*f*_*M *_(-0.10)
Background PSD	*g*_*m*_	*c*_*M *_(0.36), *c*_*m *_(0.31), *g*_*M *_(0.71), *b *(-0.10) *s*_*m *_(0.11), *C*_*M *_(0.14), *K *(-0.11), *Q*_0 _(0.16), Δ*f*_*m *_(-0.17), Δ*f*_*M *_(-0.17)
Background PSD	*c*_*M*_	*K*_*g *_(-0.60), *c*_*m *_(0.71), *g*_*m *_(0.36), *g*_*M *_(0.34), *K*_*G *_(-0.33), *C*_*M *_(-0.21), *C*_*m *_(0.21), *Q*_0 _(-0.22), Δ*f*_*m *_(0.10), Δ*f*_*M *_(0.10)

Only statistically significant (with a confidence of 99%) are shown. In parenthesis is shown the corresponding correlation value. The correlated parameters are only computed for those parameters with a measured accuracy in Table 2.

**Table 4:** Factor loadings greater than 0.5 for the first seven factors of a factor analysis with ten factors of the bootstrapped ensemble of model parameters

**Factor**	**Loadings greater than 0.5**
Factor 1	*s*_*M *_(0.98), *s*_*m *_(0.97), *K*_*s *_(0.96), *K*_*G *_(-0.75), *b *(-0.73)
Factor 2	*C*_*M *_(0.90), *G*_*M *_(0.87), *G*_*m *_(0.84), *C*_*m *_(0.72)
Factor 3	*Q*_0 _(-0.94), Δ*f*_*M *_(0.92), Δ*f*_*m *_(0.92)
Factor 4	*c*_*M *_(0.83), *c*_*m *_(0.80), *K*_*g *_(-0.76)
Factor 5	*K *(0.94), *α *(0.87)
Factor 6	*g*_*M *_(0.96), *g*_*m *_(0.74)
Factor 7	*C*_*a *_(-0.99), Δ*V/V *(0.72)

Each row shows which are the parameters correlating more than 0.5 with the factor heading the row. We show in parenthesis the corresponding correlation.

**Figure 2:**
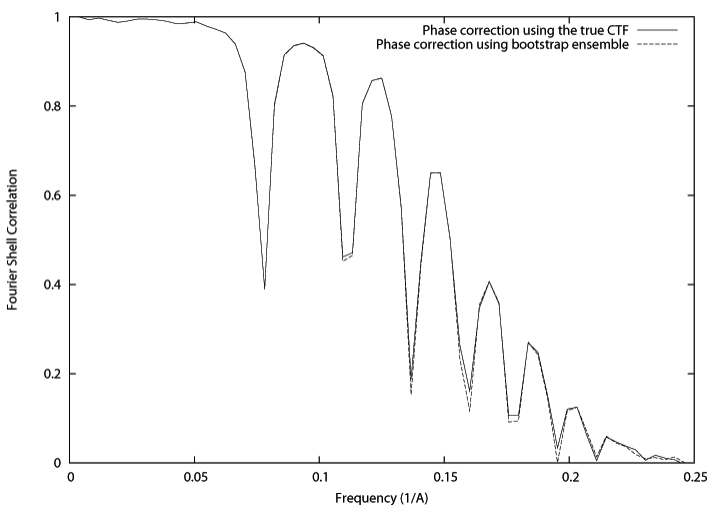
**Fourier Shell Correlation after phase correction**. Solid line: FSC of the GroEL phantom and the volume reconstructed with the phase corrected images using the truly applied CTF. Dashed line: FSC of GroEL phantom and the volume reconstructed with the phase corrected images using the bootstrapped ensemble.

**Figure 3:**
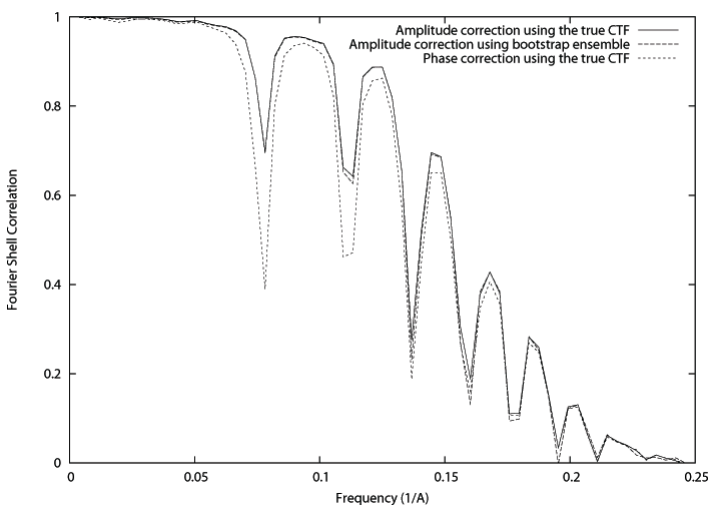
**Fourier Shell Correlation after amplitude correction**. Solid line: FSC of the GroEL phantom and the volume reconstructed with the amplitude corrected images using the truly applied CTF. Dashed line: FSC of GroEL phantom and the volume reconstructed with the amplitude corrected images using the bootstrapped ensemble. For comparison purposes, the FSC of the phase corrected volume has been added to the plot.

Finally, we used the bootstrapped ensemble to evaluate the effect of the variability of the CTF detection in the CTF correction capabilities of subsequent algorithms, particularly, those of CTF phase correction and CTF amplitude correction through IDR. For exploring this effect we used 97.4% CTFs considered to be non-failures of the CTF detection algorithm (see description of the bootstrap ensemble experiment). One of them was selected to be the true underlying CTF, while the rest were used as estimates of this true CTF. As many projections as good CTF in the ensemble were simulated of the GroEl atomic structure [20] (PDB entry code: 1GRL) with a random angular distribution, a sampling rate of 2 Å/pixel. Noise was added with a final Signal-to-Noise Ratio (SNR) of 1/3 (see Fig. 1). We corrected the CTF phase using the truly applied CTF and its pretended bootstrap estimates (one different estimate for each projection). We compared the Fourier Shell Correlation (FSC, [21]) of the volume reconstructed out of each one of these different corrections with respect to the atomic model (see Fig. 2). We also corrected the CTF amplitude using IDR with a relaxation factor of 1.8. After 10 iterations we did not observe any further impShould we haverovement of the FSC. The FSC of the volumes reconstructed using the truly applied CTF and the pretended bootstrap estimates are shown in Fig. 3. Note that in this experiment we used a single defocus value in the set of images used for reconstruction. This was done with the aim of isolating the effect of the miscorrected phase flips. The use of more defoci in the dataset would have translated in results more difficult to interpret since the zeros of one defocus group would have been masked by other defocus groups having a larger CTF value at that frequency. It is expected that if the effect of miscorrecting the phase of a single defocus groups is negligible, the combined effect of miscorrecting each defocus group independently is also negligible on the final reconstruction.

### Discussion

From the experiments performed, it turns out that the most important parameter when dealing with the CTF is the microscope voltage. This fact will certainly not come to a surprise to any practitioner in the field, but it clearly stress the point that small inaccuracies in its provision (and most CTF estimation algorithms rely on the user providing manually this value rather than automatically calculating it) result in large variations in the CTF related quantities. Since the CTF estimation algorithms try to fit as much as possible the experimentally observed PSD with the theoretically predicted PSD, this probably means that there is the possibility of a strong cross-talking between the microscope voltage and all the rest CTF parameters. Fortunately, the microscope readings of the voltage are valid up to a few tens of Volts (2 ppm/minute in a JEOL 3011) meaning that the accuracy in the estimation of this parameter is well below 0.01%.

The second set of most sensitive parameters are the ones modeling the defoci, which are estimated by all CTF estimation programs. Defoci alone do not allow to correct for any aberration caused by the CTF. For a 1D correction of the phase, at least *Q*_0 _is needed (which also has a relatively large weight on the sensitivity of the CTF zeros, although not as large as those of the microscope voltage and the defoci). Some programs estimate *Q*_0 _although in some other programs it is also directly input by the user. Again, due to its relatively medium sensitivity, small errors in the user estimation of *Q*_0 _probably turn into medium errors in the estimate of the zeros, or in a medium cross-talking to the other CTF parameters. As shown by the factor analysis, *Q*_0 _changes correlate well with changes in the defoci. Thus, the defoci values are strongly affected by the estimation of *Q*_0_. However, as shown by the bootstrap analysis, the accuracy of our algorithm in the estimation of the defoci values in the experiment run was in the range around 1% meaning that this estimate is rather stable. Note that *Q*_0 _has to be estimated mostly at low frequencies. In this region of the spectrum there is an important contribution of the amplitude contrast where high background arising from direct electron beam and inelastic scattering makes the estimation difficult. Therefore, it might be good to perform the estimation of *Q*_0 _by some other means [22,23]. A 2D phase correction also needs the estimation of the azimuthal angle *θ*. Although, the micrograph dataset of our experiment was not perfectly non-astigmatic, there was no micrograph with large astigmatism. This resulted in a relatively low sensitivity to *θ *in the three measured quantities. However, if strongly astigmatic images were recorded, the sensitivity to this parameter may have been much larger. The next most sensitive parameters are related to the background PSD (*s*_*m*_, *s*_*M*_, *b *and *K*_*G*_). *s*_*m*_and *s*_*M *_take care of the PSD shape at low frequencies, *K*_*G *_takes care of the medium frequency range, and *b *explains the background PSD at high frequencies. This means that it is important to do a good fitting in the whole spectrum. Of course, these parameters are only important if the full experimental PSD is to be fitted. Fitting of the background is absolutely crucial if an accurate amplitude correction is to be performed. The high sensitivity of the PSD to the background PSD highlights the importance of a good background fitting or background subtraction. Those programs that estimate the CTF zeros by first subtracting the background need to be sure that the subtracted background is not modifying the positions of the zeros. As is shown by the internal structure of the regression model revealed by bootstrap resampling, there is a significant "cross-talking" between the background parameters and the defoci. Finally, the two most important parameters of the CTF envelope decay are the chromatic aberration and the energy spread of the electrons at the source. Both parameters affect the *E*_*spread *_term that depends with the fourth power of the frequency (|**R**|^4^) and not with the second power as a Gaussian. However, the coherence envelope *E*_*coherence *_depends as a Gaussian with frequency and is governed by the sensitivity to the defoci, which is much larger than that of the chromatic aberration or the energy spread. The coefficient of |**R**|^2 ^in *E*_*coherence *_is *π*^2 ^*α*^2^|Δ*f *(**R**)|^2^, this means that the envelope is also astigmatic for astigmatic images. Any program that does not fit an astigmatic Gaussian envelope cannot properly correct for the amplitude decay of astigmatic images. On the other hand, *E*_*spread *_is not astigmatic. According to the relative sensitivities, *E*_*coherence *_is more important than *E*_*spread*_.

**Figure 4:**
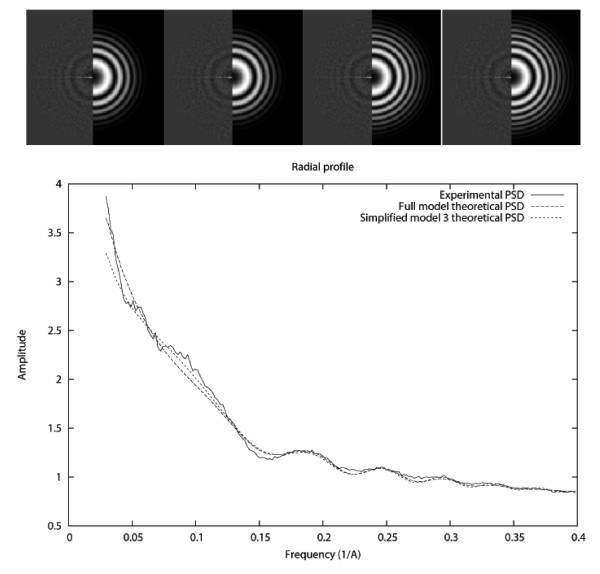
**CTF fitting examples**. Top: 2D representation of the experimental and theoretical PSDs for the Full model (left), and Simplified models 1, 2 and 3. Bottom: Radial average of the fitting for the Full model and the Simplified model 3.

On the other extreme, we have identified variables that are not so sensitive, meaning that they allow to be estimated with large errors without affecting much to our overall comprehension of the CTF effects. These are mostly related to the background (*K*_*s*_, *C*_*m*_, *K*_*g*_, *G*_*M*_, *C*_*M*_, *G*_*m*_, *c*_*m*_, *θ*_*G*_, *g*_*M*_, *g*_*m*_, *c*_*M*_, *θ*_*s*_, *θ*_*g*_) but we also found some of them related to the CTF model: the spherical aberration (*C*_*s*_), the drift (Δ*R *and Δ*F*), and the sampling rate (*T*_*m*_). Interestingly, the drifted images are usually removed from the experimental datasets, and the spherical aberration and the sampling rate are provided by the user, meaning that fortunately for these parameters, small errors in their estimation translate into small errors in the CTF. We also explored the possibility of simplifying our model to a less accurate model by removing those less sensitive variables. It turned out that the drift parameters can be safely removed, but the background negative Gaussian parameters (the next set of least sensitive parameters) cannot be removed without committing large fitting errors especially in 2D (as can be seen in Fig. 4, the Simplified models 2 and 3 show an extra Thon ring that is not present in the experimental PSD). The reason for this is that the values of *g*_*M *_and *g*_*m *_are 2,256 and 1,432 respectively. Having a low sensitivity means that you can commit small errors around the nominal values without committing large errors in the function being studied, but it does not mean that the corresponding term can be completely removed. This is confirmed by the 95% confidence intervals computed through the experimental parameter distribution estimated by bootstrapping. Since the null hypothesis that any of the regression parameters is zero has been rejected for all parameters (except the azimuthal angle), we conclude that all parameters in our model really explain a part of the PSD behavior. It is also interesting to see how a background term as the subtractive Gaussian influences the envelope parameters so that when this Gaussian is removed, the envelope is such that an extra ring is made visible.

We also explored a methodology to determine the accuracy in the estimation of each parameter. For this we made use of bootstrap resampling to build an empirical distribution of each parameter. From this distribution we were able to estimate the accuracy in each parameter. It is interesting to see that under similar fitting conditions (the accuracy of the goal function of the regression was 0.5% meaning that all the bootrstrapped models were similar in explanation power), the most important parameters are very precisely estimated (1% in the case of the defoci, and about 5% in the case of *s*_*m*_, *s*_*M *_and *b*). The rest of parameters are much less centered around a central value and can vary much more (some of them like the aperture semiangle can vary up to 54%, without affecting much the regression goal function).

The ensemble of models stemming from the bootstrap resampling also allows to identify which model parameters influence a given parameter by means of identifying statistically significant correlations. For each model parameter, a set of significantly correlated parameters is computed and shown in Tables 3 and 4. It is interesting to see that there is a non-negligible correlation between all the components of the background PSD, meaning that similar explicative power can be attained simply by shifting part of the information from one background component to the other. The sign of the correlation indicates whether a given parameter must be increased or decreased if another parameter is increased. It is also important to recognize the non-negligible correlation between the two most important parameters (defoci) and the PSD model at low frequencies (explained by *Q*_0_, the base line *b*, the term headed by *K*_*s *_and the term headed by *K*_*g*_). Most of these terms correspond to the background estimation. This implies that the background must be carefully estimated rather than simply subtracted after a rough estimation provided by a low-pass filter of the experimental PSD. A theoretical model for the background PSD is lacking in electron microscopy, and instead we use an arbitrary model that has been shown to perform well with micrographs. However, more research should be carried out in this direction to correctly identify a physically justified model for the background PSD.

The use of Factor Analysis with the bootstrapped ensemble of models allowed us to identify those main components of the regression. Seven groups of parameters were identified by keeping only those factor whose associated eigenvalue was larger than 1. These groups of parameters explain different aspects of the regression and within each group parameters are strongly correlated with each other. The groups identified were:

• Oscillatory behavior of the CTF: through the parameters *Q*_0_, Δ*f*_*M *_and Δ*f*_*m*_

• Amplitude and coherence decay of the CTF: controlled by the parameters *K *and *α*.

• Energy spread decay of the CTF: controlled by the parameters *C*_*a *_and Δ*V/V*.

• General fitting of the background PSD: particularly through the parameters *s*_*M*_, *s*_*m*_, *K*_*s *_(low-frequency), *K*_*G *_(medium-frequency), *b *(high-frequency). There is a strong "cross-talking" between all the components.

• Fitting of the background PSD at medium frequencies: through the parameters *C*_*M*_, *C*_*m*_, *G*_*M *_and *G*_*m*_.

• Fitting of the background PSD at low frequencies: with the parameters *c*_*M*_, *c*_*m *_and *K*_*g *_controlling the amplitude and location of this low frequency model.

• Fitting of the background PSD at low frequencies: with the parameters *g*_*M *_and *g*_*m*_controlling the width of this low frequency model. Note that this set of parameters is not so much correlated to the previous set controlling different features of the same part of the model.

The Factor Analysis reveals the internal structure of the cross-talking between parameters. As can be seen in the following example, cross-talking between parameters is unavoidable. Let us consider, for instance, the group formed by *C*_*a *_and Δ*V/V*. It participates exclusively in the envelope due to the beam energy spread. It can be easily seen in Eq. (7) that increases in *C*_*a *_can be compensated by decreases in Δ*V/V *and viceversa (this also explains the different signs of these two parameters with respect to Factor 7 in Table 5).

Our analysis of the effect of the variability of the CTF estimation on the CTF correction either through CTF phase correction or CTF amplitude correction shows that in the experiment performed, there is not a significant difference between the FSC of the volume corrected with the truly applied CTF and the FSC of the volume using the bootstrap ensemble. This would be pointing out that the different estimates around the true value obtained with the algorithm of [1] can be successfully used for CTF correction.

Finally, although not considered in this work, we would like to comment on the effect of the micrograph recording support (film and film scanner, or CCD camera). To the best of our knowledge, none of the CTF models published so far consider the effect of the Modulation Transfer Function (MTF) of the recording support. They are usually considered to behave as lowpass filters with a relatively flat bandpass region within which the microscopic information is supposed to fit. If the MTF actually modulated the amplitudes of the microscopic information, this would translate into variations of the areas of the CTF and the PSD analyzed in this paper, but not in variations in the positions of the zeros. This means that the MTF has no effect on the sensitivity analysis performed for the first zero of the CTF. The effect of a monotonically decaying MTF on the analysis performed in this paper would be a decrease in the overall sensitivity of all the parameters (since all the areas under the CTF and PSD would be smaller).

## Conclusion

In this article we have devised a mathematical methodology to quantitative analyze CTF models. This mathematical framework gives a clue about the sensitivity of each CTF parameter, the origin of cross-talking between parameters and which parameters are more likely to induce cross-talking. At the same time, the use of our methodology also permits the estimation of the accuracy in the determination of each CTF parameter. For the CTF and PSD model of [1] we have shown that the most important parameters are the microscope voltage and the defoci, then a few parameters determining the background PSD revealing the importance of a good background fitting, and finally *Q*_0 _(representing the mixture of amplitude and phase contrast) and the chromatic aberration so that amplitude correction can be performed.

The bootstrap analysis performed has revealed the accuracy achieved in the estimation of each parameter. Generally speaking, the most sensitive parameters identified in the previous section are estimated with higher accuracy. In particular the most important parameters, voltage and defoci, are estimated with accuracies in the order of 0.01% and 1%. The bootstrap analysis also allowed to identify the internal structure of the model (which parameters influence in which). Applying Factor Analysis to the bootstrapped data, we have been able to divide the PSD parameters into seven groups each one accounting for a different aspect of the final PSD fitting.

We have also checked that if the PSD is less sensitive to a parameter, it does not mean that it can be safely removed from the model (in fact the hypothesis tests performed with the experimental parameter distribution estimated by bootstrapping indicate that they cannot be removed from the regression without losing modeling power). It rather means that we are allowed to commit a bigger error in its estimation without affecting too much the final result. Through the estimation of the empirical joint distribution of the model parameters we have shown that the background PSD model is crucial in order to have meaningful estimates of the CTF parameters.

Finally, we have checked whether the variability observed in the CTF detection affects or not the quality of the CTF correction, either phase or amplitude correction. In our experiments, the different estimates of the CTF do not significantly hinder the posterior CTF correction algorithms.

Although we have applied the sensitivity analysis to a single CTF model, the idea is general and can be applied to other CTF models in order to reveal their most sensitive parameters as well as the internal structure of the model as described through the factor analysis and the correlation between model parameters.

## Methods

In order to make the paper self-contained we briefly summarize the CTF model of [1], and then we proceed with the sensitivity analysis and the accuracy of the CTF estimates.

### CTF model

We assume that the model of image formation in the electron microscope is

(1)

where **R **∈ ℝ^2 ^denotes the spatial frequency in Å^-1^. The structure of this PSD is formed by two terms. The first one is the PSD of the noise colored by the CTF (represented by *H *(**R**)). The second one is the PSD after CTF and is referred to as "background" PSD.

An ideal microscope has a frequency response given by

(2) *H*_*ideal *_(**R**) = -(sin(*χ *(**R**)) + *Q *(**R**) cos(*χ *(**R**))),

where *Q *(**R**) is the mixture of amplitude and phase contrast at each frequency. In the model of [1] it is assumed to be constant, *Q*_0_. *χ *(**R**) determines the shape of the sinusoidal dependency of the CTF

(3)

*C*_*s *_represents the spherical aberration coefficient. Δ*f *(**R**) is the defocus vector described by the ellipse:

(4) Δ*f *(**R**) = (Δ*f*_*M *_cos(∠**R **- *θ*), Δ*f*_*m *_sin(∠**R **- *θ*)).

∠**R **is the angle of the 2D frequency **R**. The major and minor semi-axes of the ellipse are Δ*f*_*M *_and Δ*f*_*m*_, respectively. The angle of the major semi-axis with respect to the horizontal axis is *θ*. *λ *is the electron wavelength computed as

(5)

being *V *the acceleration voltage of the microscope.

A real microscope has a frequency response that is the combination of the ideal CTF with a damping envelope *E *(**R**), which results in a low-pass filtering of the ideally projected 3D object. The model in [1] considers three different aberration sources: the beam energy spread, the beam coherence, and the sample drift. Thus, the frequency response of a real microscope is

(6) *H *(**R**) = *E*_*spread *_(**R**) *E*_*coherence *_(**R**)*E*_*drift *_(**R**) *H*_*ideal *_(**R**).

The beam energy spread envelope is computed as

(7)

where *C*_*a *_is the chromatic aberration coefficient, and  is the energy spread of the emitted electrons represented as a fraction of the nominal acceleration voltage.

The beam coherence envelope is computed as

(8) *E*_*coherence *_(**R**) = exp (-*π*^2 ^*α*^2^(*C*_*s *_*λ*^2 ^|**R**|^3 ^+ |Δ*f *(**R**)||**R**|)^2^),

where *α *is the semi-angle of aperture.

Finally, the envelope due to the sample shift is computed as

(9) *E*_*drift *_(**R**) = *J*_0 _(*π*Δ*F λ*|**R**|^2^)sinc(|**R**|Δ*R*),

where Δ*F *is the mechanical displacement perpendicular to the focal plane and Δ*R*, the displacement in the focal plane (drift).

Summarizing, the parameters required to fully specify the CTF in the model are

(10)

The formal model for the background PSD used in [1] is

(11)

where

(12)

The first term provides a constant baseline; the second term is a decaying exponential representing the background PSD behavior; the third and fourth terms of the model are intended to provide more flexibility in the PSD modeling process. All terms are assumed to be elliptically symmetric accounting for a possible anisotropy of the spectrum after convolution with the Point Spread Function (the real-space counterpart of the CTF). Parametrical models of the corresponding ellipses are given in Eq. 12. This model for the background was established purely on empirical basis without any theoretical support. To the best of the authors' knowledge there is no well-established physical model for the background noise, and the merits of the proposed models relay in their ability to fit the experimentally observed PSDs.

To summarize, all the parameters involved in the definition of the background PSD are

(13) (*b, K*_*s*_, *s*_*M*_, *s*_*m*_, *θ*_*s*_, *K*_*G*_, *G*_*M*_, *G*_*m*_, *C*_*M*_, *C*_*m*_, *θ*_*G*_, *K*_*g*_, *g*_*M*_, *g*_*m*_, *c*_*M*_, *c*_*m*_, *θ*_*g*_).

### Sensitivity analysis

The CTF function *H *(**R**) depends only on **R **assuming that the estimated CTF parameters, , are fixed. However, if we consider the CTF parameters to be also variables, then we could define a new function  (**R**, Θ) such that *H *(**R**) = (**R**, ). Because of the noise, we assume that the estimated parameters are not exactly the true parameters, Θ*, but a close approximation, ie,  = Θ* + ΔΘ, being ΔΘ a small displacement around the true parameters.

We consider now which is the error in the CTF by using the estimated parameters  instead of the true parameters Θ*. For doing so, we compute the Taylor series expansion of the function  (**R**, Θ) around the point 

(14)

We define Δ*H *(**R**) = (**R**, Θ*) - (**R**, ). For each CTF parameter, *x*, we define its variation as Δ*x *= x*- , where x* is the parameter true value and  is our estimate. With this notation, we can express Eq. 14 as

(15)

This latter equation relates the error committed in the estimation of the CTF to the error committed in the estimation of all its parameters. Errors in one parameter may compensate with errors in some other parameter. With thirteen parameters (*K*, *V*, *C*_*s*_,..., Δ*R*), the amount of possible combinations is huge. For this reason, parameters are usually studied one by one. Moreover, sensitivity analysis is not as much interested in the sign of the error as in its magnitude. For these reasons, for each parameter *x *of the CTF we study the absolute value of the error in the CTF due to an error in a single parameter:

(16)

The previous equation approximates the absolute error committed in the CTF value when a given absolute error in the estimation of *x *is committed. However, it would be more interesting to compute relative errors of the CTF with respect to relative errors in *x*. Therefore, we modify our previous absolute error estimate to a relative error estimate:

(17)

Eq. 17 expresses the relative error in the CTF as a function of the relative error in the parameter estimate. Calling *S*_*H*_(, **R**) to , we have

(18)

ie, *S*_*H *_(, **R**) represents the sensitivity of the CTF at frequency **R **to variations in the parameter *x*, specifically when its value is . Solving for *S*_*H *_(, **R**) we have

(19)

Eq. 19 provides the sensitivity at a single frequency **R**. In order to obtain a single numerical observer that reflects the overall sensitivity over the whole CTF, we average the sensitivity over the square , where **R**_*s *_is the sampling rate in Å^-1^:

(20)

*S*_*H *_() is a measure of the overall error sensitivity of the CTF at a particular estimate of the parameter, . However, an ensemble of micrographs may have different values of the same parameter. Therefore, it is more reasonable to have an average sensitivity assuming that the parameter  is in fact a random parameter with an underlying distribution that can be estimated from the micrograph ensemble.

(21)

where *E*{·} is the expectation operator with respect to the distribution of .

We propose to use  to sort all CTF parameters according to their sensitivity. Parameters with low sensitivity may be estimated more roughly while the estimation of more sensible parameters has to be more careful. The sensitivity also reflects indirectly which are the most important parameters defining the characteristics of a given CTF. The more sensitive is a given parameter, the more important it is to estimate it correctly.

### Accuracy of the CTF estimates

The problem solved in [1] can be regarded as a regression problem of the experimentally observed PSD as a function of the frequency. The model parameters are given by the PSD parameters described in the previous section. For determining the accuracy of each parameter in the model, an empirical distribution of each parameter can be constructed through bootstrap resampling of the measured data (the pairs frequency-experimental PSD) [24]. For each resampled dataset, the PSD model parameters are estimated producing, thus, an ensemble of parameter estimates out of which the empirical distribution of each parameter is easily estimated. An important consequence of bootstrap resampling is that the distribution of the model parameters of the resampled datasets around the model parameters estimated from the whole dataset is the same as the distribution of the model parameters from the whole dataset around the true parameters. This allows to estimate many statistics of the unknown distribution of the model parameters estimated from the whole dataset from the bootstrapped distribution. In particular, we concentrate on two aspects: the estimation of the accuracy of each model parameter (computed as the percentage of variation of that parameter with respect to its nominal value, ||); and the computation of the confidence interval for each model parameter to test the hypothesis that each one is significantly different from zero (if they are, they cannot be removed from the model without losing part of the modeling power).

The empirical joint distribution of all parameters can also be computed using bootstrapping, and it can be used to estimate the possible cross-talking between model parameters through the computation of the correlation matrix from the bootstrapped ensemble. Statistically significant correlations show which parameters have an influence on other parameters: the larger the correlation coefficient in absolute value, the stronger the influence. In this way, for any model parameter we can construct a list of other variables in the model influencing it.

Careful observation of the influence lists easily pinpoints groups of variables where all of them influence all the others, as shown in the Results Section. However, it is not straightforward to manually identify these variable groups. For this purpose, we propose the use of factor analysis [25] to identify the underlying factors explaining the bootstrapped ensemble. The elements of the loading matrix provide an estimate of the correlation between the model parameters and the identified factors. Only statistically significant correlations are considered. As shown in the Results Section, each factor mainly correspond to a group of variables that are strongly interrelated plus a few of low correlated, although significantly, variables.

## Authors' contributions

COSS designed and implemented most of the experiments. AO automated part of the experiments and EMO performed the experiments and organized the results. JC revised the manuscript and provided useful suggestions during the tests.
